# Revolutionizing Pediatric Endodontics: A Two-Decade Narrative on the Evolution, Efficiency, and Future of Pediatric Rotary Systems

**DOI:** 10.7759/cureus.97892

**Published:** 2025-11-26

**Authors:** Antra Singh, Sonali Saha, Kavita Dhinsa, Saumya Navit, Karishma Jaiswal, Mitusmita Kalita, Arunima Sarkar, Shreya Pandey, Seema Gupta

**Affiliations:** 1 Department of Paediatric and Preventive Dentistry, Sardar Patel Post Graduate Institute of Dental and Medical Sciences, Lucknow, IND; 2 Department of Paediatric and Preventive Dentistry, Saraswati Dental College and Hospital, Lucknow, IND; 3 Department of Orthodontics, Kothiwal Dental College and Research Centre, Moradabad, IND

**Keywords:** endodontics, files, pediatric, review, rotary

## Abstract

Preservation of primary teeth plays a vital role in maintaining arch integrity and guiding the eruption of permanent successors. Pulpectomy remains the treatment of choice for retaining non-vital primary teeth; however, traditional manual instrumentation is often time-consuming and technically demanding because of the complex and resorbing root morphology. Over the past decades, the advent of pediatric-specific nickel-titanium (NiTi) rotary systems has revolutionized endodontic practice in children by offering enhanced precision, safety, and efficiency. This narrative review synthesizes the developmental trajectory, design innovations, metallurgical advancements, and clinical performance of rotary file systems specifically designed for primary teeth. The literature, drawn from major databases spanning 2000-2025, highlights the transformation from modified adult files to pediatric-optimized systems, such as Kedo-S (Kedo Dental, Chennai, India), Kedo-SG Blue (Kedo Dental), Pro AF Baby Gold (Dentobizz, Yavatmal, India), and Fanta AF™ (Fanta Dental Materials, Shanghai, China), which integrate shorter lengths, variable tapers, and heat-treated or nano-coated NiTi alloys. These systems have demonstrated superior canal shaping, reduced instrumentation time, improved obturation quality, and greater patient cooperation than conventional hand files. Advancements in metallurgy, including controlled memory, gold and blue heat treatments, and nanocoating, have markedly improved file flexibility, fatigue resistance, and cutting control. However, challenges regarding technique sensitivity, cost, and training requirements persist. This review underscores the importance of adhering to precise torque and speed parameters, ensuring correct working length determination, and maintaining irrigation safety to avoid procedural mishaps. Emerging trends, such as artificial intelligence (AI)-assisted imaging, nanotechnology-driven coatings, reciprocating motion systems, and 3D-printed simulation training, promise to further refine future pediatric rotary endodontics. Collectively, these innovations represent a shift toward safer, faster, and more predictable pulpectomy outcomes. Rotary instrumentation has evolved from the adaptation of adult techniques to a specialized, evidence-based cornerstone of contemporary pediatric dental care.

## Introduction and background

The preservation of primary teeth until their natural exfoliation is essential for maintaining arch integrity, guiding the eruption of permanent successors, and ensuring optimal oral function in children. Premature loss of these teeth due to caries or pulp pathology can disrupt occlusal development, cause space loss, and promote deleterious oral habits, leading to malocclusion [1]. Pulpectomy remains the treatment of choice for maintaining non-vital primary teeth; however, traditional manual instrumentation using stainless steel files often poses challenges owing to the complex, tortuous, and resorbing canal morphology typical of primary roots [2]. These limitations, coupled with young patients’ limited cooperation and increased procedural time, have historically deterred clinicians from performing pediatric endodontic therapy.

The advent of nickel-titanium (NiTi) rotary instrumentation has introduced a paradigm shift in endodontics [2,3]. Initially developed for permanent teeth, rotary systems offer greater flexibility, shape memory, and superior cleaning ability, minimizing procedural errors, such as ledging or canal transportation [4]. Recognizing the need for child-specific adaptations, pediatric rotary systems such as Kedo-S (Kedo Dental, Chennai, India), Kedo-SG Blue (Kedo Dental), Pro AF Baby Gold (Dentobizz, Yavatmal, India), and Fanta AF™ (Fanta Dental Materials, Shanghai, China) have been designed with shorter working lengths, variable tapers, and controlled memory (CM) alloys to accommodate the anatomical and physiological characteristics of deciduous roots [5,6].

Contemporary studies have demonstrated that pediatric rotary systems significantly reduce instrumentation time, improve obturation quality, and enhance patient cooperation compared to conventional hand files [2-5,7]. The integration of advanced metallurgical technologies, such as blue-heat-treated and nano-coated NiTi alloys, has further improved cyclic fatigue resistance and operational safety [8]. Despite their higher cost and technique sensitivity, these systems have redefined the standards of pediatric endodontic care through enhanced precision, reduced chairside time, and superior canal-shaping outcomes [8,9]. This narrative review aims to critically synthesize two decades of literature on pediatric rotary file systems, highlighting their evolution, design innovations, and comparative clinical performance across in vitro, in vivo, and combined studies, with an emphasis on the Kedo-S series and its successive generations as benchmarks in modern pediatric endodontics.

## Review

Search methodology

**Figure 1:**
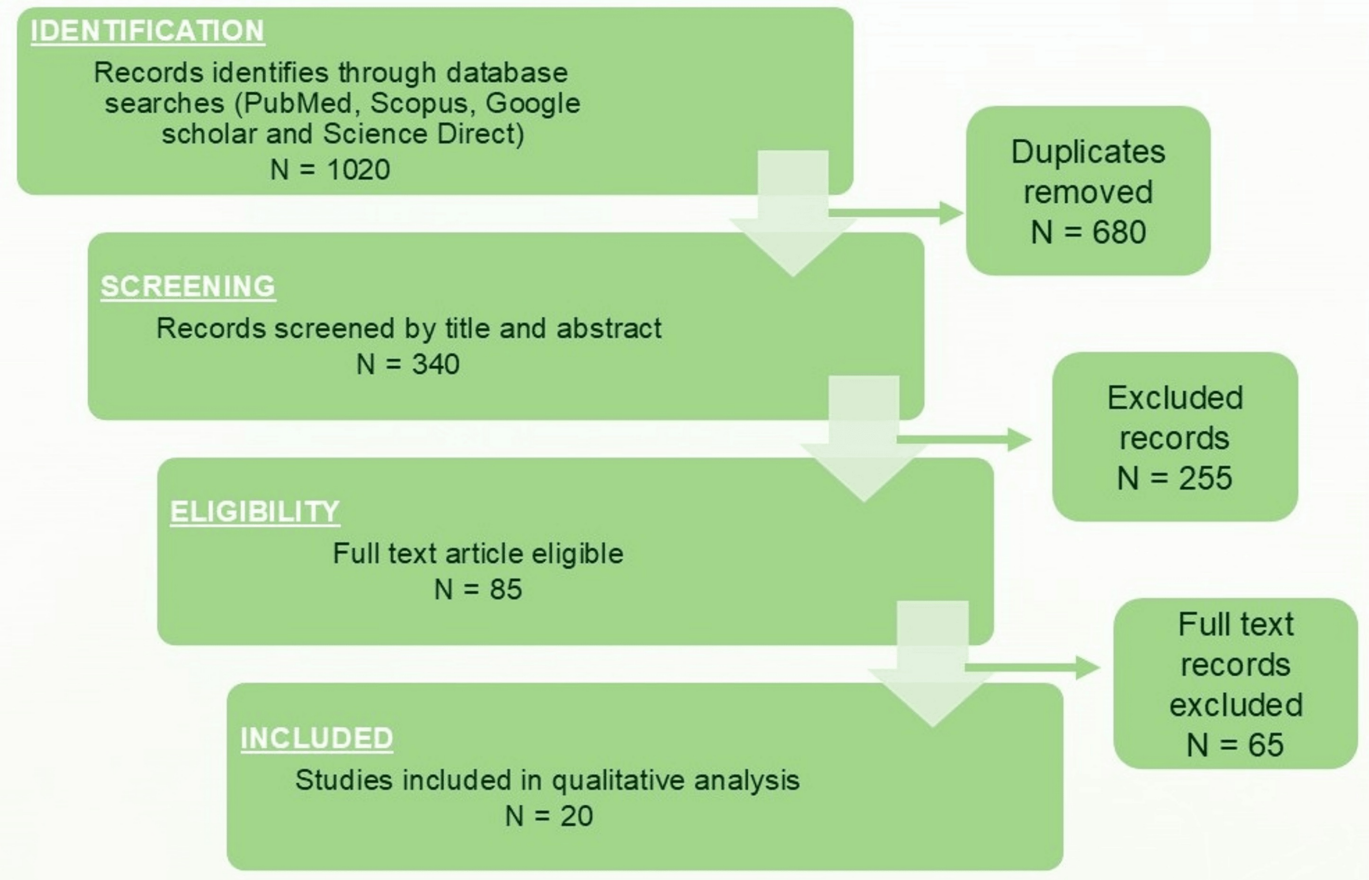
PRISMA chart for the included study. This image was created using Microsoft^®^ PowerPoint^®^ 2021 MSO (Version 2510 Build 16.0.19328.20144) (Microsoft Corp., Redmond, WA, US) PRISMA: Preferred Reporting Items for Systematic reviews and Meta-Analyses

A comprehensive literature search was conducted to gather relevant studies, reviews, and clinical trials pertaining to rotary instrumentation in pediatric endodontics (Figure 1). Electronic databases, including PubMed, Scopus, ScienceDirect, and Google Scholar, were systematically searched for publications from January 2000 to September 2025. The search was performed using a combination of Medical Subject Headings (MeSH) and free-text keywords such as “pediatric rotary endodontics,” “primary teeth pulpectomy,” “NiTi rotary files,” “Kedo-S,” “Pro AF Baby Gold,” “Fanta AF,” and “pediatric rotary instrumentation.” Boolean operators (“AND,” “OR”) were used to refine the search and ensure comprehensive coverage.

Only studies published in English were included in this meta-analysis. The search encompassed in vitro, in vivo, ex vivo, and finite element analysis (FEA) studies as well as narrative and systematic reviews, randomized controlled trials, and product evaluations focusing on pediatric rotary file systems. Articles on rotary instrumentation in permanent teeth, case reports without experimental comparisons, and non-dental studies were excluded.

Titles and abstracts were screened to assess their relevance, followed by a review of full-text articles that met the inclusion criteria. The reference lists of key articles were also manually searched to identify additional pertinent studies. Data were extracted and synthesized narratively, emphasizing the evolution, design features, metallurgical advancements, clinical outcomes, advantages, limitations, and future perspectives of pediatric rotary systems, with particular attention to the Kedo series and its comparative performance with other systems, such as Pro AF Baby Gold, and Fanta AF™.

History

The concept of rotary instrumentation dates back to the late 19th century, when Oltramare introduced mechanical instruments for root canal shaping. However, it was not until the development of the NiTi alloy by Walia et al. in 1988 that rotary systems became clinically viable [10]. NiTi files offer unique superelasticity and shape memory, allowing better adaptation to curved canals, while minimizing canal transportation and procedural errors.

The first application of NiTi rotary files in primary teeth was reported by Barr et al. in 1999 [11], who used the ProFile system (Dentsply Sirona, Charlotte, NC, USA) in pulpectomy procedures. Early attempts with adult rotary systems, such as ProTaper (Dentsply Sirona), Mtwo (Dentsply Sirona), and Hero Shaper (Micro-Mega, Besançon, France), demonstrated improved cleaning and shaping efficiency, but were limited by excessive taper and length unsuitable for primary dentition [12,13]. These systems often cause overpreparation and extrusion of debris due to the delicate and resorbing nature of the primary roots.

The introduction of pediatric-specific systems marked a turning point. Jeevanandan in 2017 [5] introduced the Kedo-S rotary file, which was the first file exclusively designed for primary teeth. This innovation was followed by successive generations, Kedo-SG, Kedo-SG Blue, Kedo-S Square, Kedo-S Plus, and Kedo-S Nano Plus, each addressing previous limitations through improved metallurgy, variable taper, and adaptive geometry [6]. Parallel advancements, such as Pro AF Baby Gold and Fanta AF™, further diversified options for clinicians [14,15].

**Figure 2:**
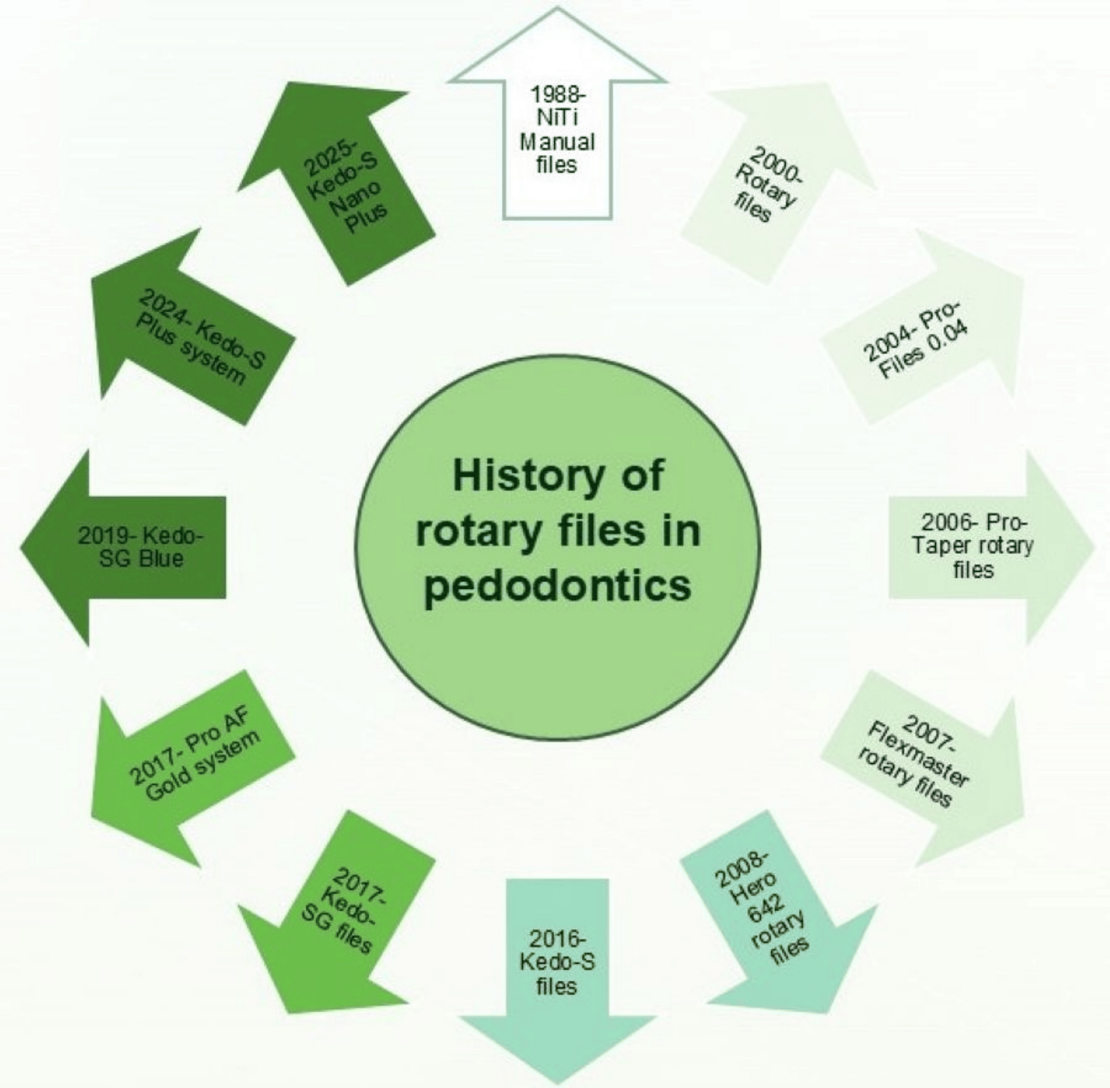
History of pediatric rotary files. Image credit: Dr. Sonali Saha This image was created using Microsoft^®^ PowerPoint^®^ 2021 MSO (Version 2510 Build 16.0.19328.20144) (Microsoft Corp., Redmond, WA, US) NiTi: nickel-titanium

By 2024, FEAs and in vitro testing had validated the mechanical superiority of these pediatric-specific files, confirming enhanced torsional strength, cyclic fatigue resistance, and efficient canal shaping with minimal dentinal damage [16,17]. The chronological evolution of these systems reflects a progressive shift from the modification of adult instruments to precision-engineered designs tailored to pediatric endodontic requirements (Figure 2).

Design characteristics and metallurgical advancements

The clinical success of rotary endodontic files is largely determined by their design features, specifically taper, cross-sectional geometry, tip configuration, and alloy composition [6]. Pediatric rotary systems have been developed with these critical parameters to ensure effective cleaning and shaping, while minimizing the risk of structural damage to the thin and resorbing roots of primary teeth [7]. NiTi alloys, which are the cornerstone of these systems, provide exceptional superelasticity and shape memory, allowing instruments to follow curved canals without permanent deformation. Modern pediatric file systems incorporate advanced metallurgical treatments, such as CM, M-wire, and blue or gold thermal modifications, which significantly enhance flexibility, resistance to cyclic fatigue, and overall durability [18]. For instance, the blue oxide coating used in Kedo-SG Blue files improves surface hardness and corrosion resistance, thereby extending the operational lifespan of the file [19].

To adapt to the anatomical constraints of the primary teeth, pediatric rotary systems are designed with shorter overall lengths, typically 16-18 mm, with working portions ranging from 10 to 12 mm. They also exhibit variable tapers (0.25-0.08), ensuring conservative canal shaping that maintains the original anatomy and minimizes the risk of overinstrumentation or root perforation [6]. The tip design and cross-sectional geometry further influence instrument performance. Non-cutting or rounded tips, as used in the Kedo and Pro AF Baby Gold systems, reduce the likelihood of apical perforation, while triangular or asymmetrical cross-sections promote efficient debris removal and lower torsional stress [20]. The Kedo-S Square generation introduced a dual cross-sectional design, triangular at the tip for effective cutting and rectangular at the coronal end for enhanced flexibility, resulting in a balanced combination of cutting efficiency and safety [21].

Rotary systems for primary teeth operate under optimized mechanical conditions, typically at speeds of 250-350 rpm and torque values of 1.5-2.4 Ncm, to account for the thinner dentinal walls and reduced resistance of primary roots [22]. The metallurgical evolution from the conventional rigid NiTi of the original Kedo-S to the blue heat-treated and nano-coated variants, such as Kedo-SG Blue and Kedo Nano Plus, illustrates continuous refinement aimed at improving instrument longevity, flexibility, and cutting control [6,22]. Collectively, these progressive advancements in design and metallurgy represent a synergistic evolution of biomechanics and pediatric ergonomics, ultimately enhancing procedural safety, efficiency, and predictability in pediatric endodontic practice.

Clinical efficiency and procedural advantages

Numerous studies have consistently demonstrated that rotary instrumentation offers superior clinical efficiency and procedural advantages in pediatric endodontics compared with conventional manual filing techniques [2-4,7]. One of the most significant benefits is the substantial reduction in instrumentation time by nearly 40%-70%, which is particularly advantageous when treating young, anxious, or uncooperative children with limited attention spans [7,13,15]. Amorim et al. [23] reported that the HyFlex EDM^®^ rotary system (Coltène/Whaledent Inc., Cuyahoga Falls, OH, US) required significantly less time for canal preparation than manual K-files. Although HyFlex EDM is primarily designed for permanent teeth, Amorim et al. used it in primary molars because its high flexibility, CM, and superior cutting efficiency allow safe adaptation in primary canals. In their study, these properties enabled faster and more efficient canal preparation compared with manual K-files. Kohli et al. [7] observed that the Kedo-SG Blue system achieved optimal obturation in the shortest duration among the tested rotary systems. The reduction in clinical time not only enhances efficiency but also improves patient cooperation and overall procedural success.

In addition to time efficiency, the quality of obturation is markedly improved with rotary systems. Radiographic assessments have revealed that canals instrumented with rotary files exhibit more uniform obturation with fewer voids than those prepared manually [7]. This improvement can be attributed to the controlled and consistent taper produced by rotary files, which enhances the adaptation of obturating materials, such as calcium hydroxide-iodoform pastes, to the canal walls, ensuring better sealing and long-term success.

The effectiveness of rotary systems in cleaning and shaping has also been supported by scanning electron microscopy (SEM) analyses, which show that they remove debris and smear layers more efficiently than hand files [2]. Moreover, rotary instrumentation preserves the canal anatomy by maintaining natural curvature and minimizing procedural errors such as ledging, zipping, or canal transportation. The continuous rotational motion and flexibility of NiTi instruments allow for uniform dentin removal without excessive thinning of the canal walls, thereby preserving the structural integrity of the tooth [6].

Comparative evaluation: rotary vs. manual instrumentation

Multiple in vitro and clinical studies have compared rotary and manual instrumentation techniques for primary teeth [2-5]. A previous study showed that rotary files produced a more conical preparation and better cleaning efficacy than hand K-files in primary teeth [24]. Silva et al. [4] observed a significant reduction in instrumentation time using rotary systems. Similarly, Mohamed et al. [21] demonstrated that both the Kedo-S Square systems showed a good taper in the maximum number of root canals, compared to conventional manual K- and H-files.

Amorim et al. [23] compared HyFlex EDM^®^ with manual K-files in a 12-month follow-up and found that both achieved satisfactory clinical outcomes; however, rotary files offered significantly faster preparation. Kohli et al. [7] and Jeevanandan [5] confirmed the superior obturation quality and efficiency of the Kedo systems. In vitro analyses using micro-CT and finite element modeling have further demonstrated that rotary systems provide greater canal centricity, uniform taper, and less dentin removal [14,16,17]. These findings consistently indicate that pediatric rotary systems outperform manual techniques in terms of efficiency, reproducibility, and biomechanical safety without compromising cleaning ability.

In addition to mechanical and clinical outcomes, rotary instrumentation offers behavioral advantages in pediatric settings. A significant reduction in chairside time minimizes patient discomfort, enhances cooperation, and fosters a more positive dental experience, which is crucial for establishing trust and compliance in young patients [22]. Collectively, these findings underscore the multifaceted advantages of pediatric rotary systems in achieving superior cleaning, shaping, and obturation quality within shorter procedural durations, thereby optimizing both the clinical outcomes and patient management.

Pediatric-specific rotary systems

**Figure 3:**
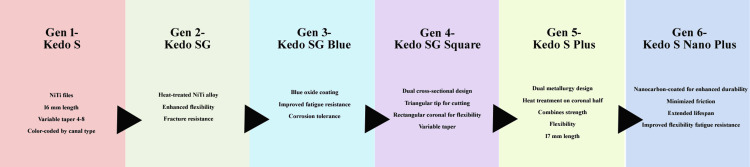
Evolution of Kedo-S rotary files. Image credit: Dr. Sonali Saha This image was created using Microsoft^®^ PowerPoint^®^ 2021 MSO (Version 2510 Build 16.0.19328.20144) (Microsoft Corp., Redmond, WA, US) NiTi: nickel-titanium

The emergence of pediatric-specific rotary systems has revolutionized endodontic procedures in primary teeth, with several innovative designs tailored to address anatomical and clinical challenges unique to pediatric dentistry (Figure 3). The Kedo series, introduced by Jeevanandan [5], represents the first rotary file system developed exclusively for primary teeth and has since evolved through multiple generations, each incorporating significant design and metallurgical refinements. The Kedo-S, a first-generation system, consists of NiTi files with a total length of 16 mm and variable tapers ranging from 4% to 8%, color-coded according to canal type for ease of selection. The second-generation Kedo-SG introduced heat-treated NiTi alloy, enhancing flexibility and reducing fracture risk, while the third-generation Kedo-SG Blue incorporated a blue oxide coating over a CM wire, significantly improving fatigue resistance and corrosion tolerance [6,22]. The Kedo-S Square, representing the fourth generation, features a dual cross-sectional design-triangular at the tip for effective cutting and rectangular at the coronal end for enhanced flexibility-combining cutting efficiency with safety during canal preparation [21]. More recently, the Kedo-S Plus and Kedo-S Nano Plus systems (fifth and sixth generations) have adopted nanocarbon coating technology to enhance durability, minimize surface friction, and extend the instrument lifespan [25]. FEAs have demonstrated that the Kedo-SG and Square variants exhibit uniform stress distribution under torsional load, reducing the risk of file separation and ensuring a consistent performance [16].

Another prominent innovation is the Pro AF Baby Gold system developed by Dentobizz, which employs gold heat treatment and a CM NiTi alloy. This configuration enhances flexibility, cyclic fatigue resistance, and centricity during instrumentation [15]. Cone-beam computed tomography (CBCT) evaluations have shown superior apical shaping and canal conicity with Pro AF Baby Gold compared with the first-generation Kedo-S system [15]. Similarly, the Fanta AF™ rotary system incorporates an asymmetrical cross-sectional design and a heat-treated NiTi alloy, offering efficient debris removal and enhanced flexibility. Clinical studies have reported consistent obturation quality and minimal procedural errors when using the Fanta AF™ [14]. Although initially designed for permanent teeth, it has been effectively adapted for pediatric molars. Electro-discharge machining (EDM) technology provides exceptional flexibility, controlled shaping, and resistance to cyclic fatigue, making it suitable for managing curved and constricted canals in deciduous teeth [26].

In addition to these established systems, newer pediatric rotary file systems such as Prime Pedo (Sky International Enterprises, Navi Mumbai, India), DXL-Pro (Kraft Marketing, Mumbai, India), Baby Blue (A to Z Kids Dental, India), PedoFlex (Orikam Health Care, India), and Endogal Kids (Galician Endodontics Company, Lugo, Spain) have entered the market, offering diverse cross-sectional geometries, heat treatments, and motion types, including rotary and reciprocating actions [6]. These variations allow clinicians to select systems tailored to case-specific anatomical complexities and operator preferences. Overall, the progressive refinement of pediatric rotary systems from the pioneering Kedo series to recent entrants illustrates the growing emphasis on ergonomics, metallurgy, and biomechanical precision. Each advancement has contributed to making rotary instrumentation safer, more predictable, and more efficient, thereby setting new standards for pediatric endodontic care.

Safety, limitations, and technical considerations

Despite the numerous advantages offered by pediatric rotary systems, several safety concerns, technical limitations, and procedural considerations must be acknowledged to ensure successful and predictable clinical outcomes. One of the most critical aspects is technique sensitivity, as rotary instrumentation demands strict adherence to recommended torque and speed parameters. Applying excessive force or using inappropriate motor settings can increase the risk of file separation, canal distortion, or overpreparation, particularly in the delicate and narrow root canals of primary teeth [6]. Equally important is the risk of overinstrumentation, which is heightened by the characteristic root resorption and short canal length of deciduous teeth. Therefore, accurate determination of the working length is essential to prevent extrusion of debris, irrigants, or obturating material beyond the apex, which may cause periapical irritation or damage to the underlying permanent tooth germ [27].

Although the use of modern controlled memory (CM) and blue-heat-treated NiTi alloys has significantly reduced the likelihood of file separation, this complication can still occur, especially in severely curved or constricted canals [28]. Another study stated that the application of permanent tooth protocols to primary molars may lead to lateral perforation of the inner root surface, especially in curved roots [22,24]. Another practical limitation involves economic and training barriers, as the initial costs of rotary motors and proprietary file systems may restrict their use in resource-limited clinical settings. Furthermore, adequate hands-on training and familiarity with rotary protocols are indispensable for safe and efficient operation of these systems [22].

Irrigation control also plays a crucial role in the safety of rotary endodontic procedures. Given the proximity of developing permanent tooth buds, the use of low-concentration sodium hypochlorite (1%-1.5%), followed by thorough saline irrigation, is recommended to minimize cytotoxic risks and prevent periapical irritation [22]. Despite the high short-term success rates and improved procedural efficiency, evidence on the long-term outcomes of primary teeth treated with rotary systems remains limited, particularly concerning their retention until natural exfoliation and the impact on permanent successors. Overall, meticulous attention to key procedural aspects, accurate working-length determination, controlled torque and speed, gentle instrumentation, effective irrigation, and proper obturation are fundamental for maximizing the safety and effectiveness of rotary endodontics in pediatric patients [29].

Future directions

The field of pediatric rotary endodontics continues to advance rapidly, driven by technological innovations and a growing emphasis on precision, safety, and clinician training. One of the most promising developments is the integration of artificial intelligence (AI) and digital imaging into endodontic diagnostics and treatment planning [30]. AI-assisted CBCT analysis enables a more accurate assessment of canal morphology, working-length determination, and curvature prediction, thereby minimizing the risk of procedural errors and enhancing treatment efficiency [31]. Similarly, advances in nanotechnology have transformed instrument design and performance. The application of nano-coatings has significantly improved surface hardness, wear resistance, and cutting efficiency, contributing to longer file lifespans and reduced friction during instrumentation [32].

The evolution of reciprocating motion systems represents another frontier of pediatric rotary endodontics. By combining the benefits of both rotary and reciprocating movements, these hybrid systems can reduce cyclic fatigue, minimize file separation, and maintain canal centricity, making them particularly advantageous for clinicians managing complex or curved root canals in primary teeth [33]. Meanwhile, 3D printing and simulation-based education have emerged as transformative tools for pre-clinical training and skill development. Digital platforms that utilize 3D-printed models of primary teeth enable standardized operator education, allowing students and practitioners to practice rotary instrumentation techniques under realistic anatomical conditions before performing clinical procedures [34].

Continued innovation in pediatric-specific handpieces, torque-controlled motors, and evidence-based treatment protocols is expected to further refine the safety and consistency of rotary endodontic procedures. The integration of these technological and educational advancements will not only streamline clinical workflows but also enhance patient comfort and outcomes. Collectively, these developments signify a forward trajectory toward establishing rotary instrumentation as the universal standard of care in pediatric endodontics, combining precision engineering with digital intelligence to achieve minimally invasive, predictable, and child-friendly dental care. The findings of the narrative review have been summarized in Table 1.

**Table 1:** Summary of key findings from the narrative review on pediatric rotary endodontic systems (2000-2025) CBCT: cone-beam computed tomography, RCT: randomized controlled trial, NiTi: nickel-titanium; WL: working length; EDM: electro-discharge machining; Kedo-S, SG, SH, Square: pediatric rotary file systems designed for primary teeth; cyclic fatigue: repetitive stress testing simulating file use during instrumentation

Authors [Ref. No.]	Year	Study type	Sample/teeth	Systems/comparators	Key outcomes assessed	Main findings (concise summary)
Barr et al. [11]	1999	Review article	Primary teeth	Early NiTi rotary (ProFile)	Feasibility; safety	Demonstrated clinical feasibility of NiTi rotary use in primary teeth; established foundation for pediatric-specific systems.
ElAyouti et al. [27]	2001	In vitro	Primary teeth	NiTi rotary	Working length accuracy	Found overinstrumentation risk; cited for cautious WL control in pediatric pulpectomy.
Silva et al. [4]	2004	In vitro	Primary molars	Rotary vs. manual K-files	Cleaning capacity; instrumentation time	Rotary instruments provided improved canal cleaning and significantly reduced instrumentation time vs. hand files.
Canoglu et al. [12]	2006	In vitro	Primary molars	Conventional vs. rotary vs. ultrasonic	Cleaning efficiency; obturation quality	Rotary and ultrasonic techniques enhanced cleaning efficiency compared to conventional hand instrumentation.
Azar et al. [13]	2012	In vitro	Primary teeth	Mtwo/ProTaper vs. manual	Cleaning efficiency	Rotary systems improved cleaning versus manual files but highlighted limitations due to shorter roots and taper variation.
Pirani et al. [26]	2016	In vitro	HyFlex EDM^®^	EDM-machined NiTi	Metallurgy; surface analysis	EDM process improved file strength and cyclic fatigue resistance, supporting clinical efficiency.
Jeevanandan [5]	2017	Case report/introduction	Primary teeth	Kedo-S	Feasibility; performance	Introduced Kedo-S as the first pediatric-specific NiTi rotary file with shorter taper and length.
Alfouzan and Jamleh [28]	2018	Retrospective study	Root canal-treated cases	NiTi rotary	Instrument fracture risk	Identified fracture incidence and technique sensitivity-emphasizing cautious use in pediatric canals.
Sruthi et al. [19]	2021	Double-blinded RCT	Primary mandibular molars	Kedo-SG Blue, Kedo-SH vs. manual K-files	Obturation quality; instrumentation time	Both Kedo systems showed superior obturation quality and shorter procedure time compared to manual techniques.
Rathi et al. [20]	2021	In vitro	Primary molars	Two pediatric rotary files	Cleaning efficiency; apical extrusion	Rotary systems improved canal cleaning while maintaining controlled debris extrusion.
Amorim et al. [23]	2022	RCT	Primary molars	HyFlex EDM^®^ vs. manual K-files	Instrumentation time; clinical success	Rotary files achieved similar clinical outcomes with significantly reduced preparation time.
Mohamed et al. [21]	2022	CBCT in vitro	Primary anterior teeth	Kedo-S Square	Canal shaping ability	Demonstrated precise taper formation and effective canal centering with Kedo-S Square.
Kohli et al. [7]	2023	In vivo comparative	Pediatric pulpectomies	Kedo-SG Blue and other pediatric rotary systems	Obturation quality; instrumentation time	Kedo-SG Blue achieved optimal obturation and minimal instrumentation time.
El-Desouky et al. [14]	2024	CBCT in vitro	Primary anterior teeth	Kedo-S Square; Fanta AF™ Baby; manual K-files	Shaping ability (CBCT metrics)	Pediatric rotary systems achieved superior canal shaping and centering ability compared with hand instrumentation.
Surme et al. [18]	2024	In vitro, fatigue analysis	EasyInSmile X-Baby, Scope miniScope, EndoArt Pedo Blue, and EndoArt Pedo Gold	NiTi pediatric rotary systems	Cyclic fatigue resistance	Heat-treated pediatric rotary systems exhibited superior cyclic fatigue resistance compared with conventional designs.
Manivannan et al. [16]	2024	Finite element analysis	Simulated primary roots	Pediatric rotary systems	Bending stress; torsional resistance	Finite element modeling revealed favorable stress distribution in newer pediatric rotary systems.
Musale and Mujawar [24]	2024	In vitro (CBCT)	Primary molars	Rotary vs. hand files	Canal preparation; shaping quality	Rotary systems produced more conical and centered preparations compared with hand files.
Suresh et al. [25]	2024	Observational overview	Primary molars	Multigenerational Kedo systems	Clinical outcomes; efficiency	Summarized clinical performance across Kedo generations; emphasized shorter time and safe use.
Monika Sri et al. [17]	2025	Finite element analysis	Simulated primary molars	Pediatric rotary files	Cyclic fatigue modeling	Demonstrated enhanced cyclic fatigue resistance in heat-treated pediatric files.
Swaminathan et al. [6]	2025	Scoping review	Literature 2000–2025	Pediatric rotary systems	Design evolution; clinical adaptation	Summarized 20 years of pediatric rotary evolution; highlighted safety improvements in Kedo file generations.

## Conclusions

Over the past decades, rotary endodontics has transformed the landscape of pediatric dental care. The transition from manual SS files to advanced pediatric-specific NiTi rotary systems enhanced the precision, safety, and efficiency of pulpectomy procedures. The Kedo-S series, along with systems such as Pro AF Baby Gold and Fanta AF™, exemplify innovation through optimized length, taper, and alloy design. Clinical evidence consistently demonstrates that pediatric rotary files significantly reduce instrumentation time, improve obturation quality, and preserve the canal morphology. Metallurgical advancements, especially CM and heat-treated NiTi alloys, have improved the cyclic fatigue resistance and operator safety. Despite limitations related to cost, technique sensitivity, and training, these systems represent a major advancement toward minimally invasive, predictable pediatric endodontics. Future research should focus on long-term clinical outcomes, bioengineering refinements, and integration of digital technologies to enhance standardization and accessibility. Rotary instrumentation, once a novelty in pediatric dentistry, is now an indispensable component of modern pulpectomy protocols, revolutionizing both technique and treatment experience for the clinician and the patient alike.
